# Thiol-Reactive Star Polymers Functionalized with Short Ethoxy-Containing Moieties Exhibit Enhanced Uptake in Acute Lymphoblastic Leukemia Cells

**DOI:** 10.2147/IJN.S220326

**Published:** 2019-12-11

**Authors:** Narges Bayat, Nathan McOrist, Nicholas Ariotti, May Lai, Keith CS Sia, Yuhuan Li, James L Grace, John F Quinn, Michael R Whittaker, Maria Kavallaris, Thomas P Davis, Richard B Lock

**Affiliations:** 1 Leukemia Biology Program, Children’s Cancer Institute, Lowy Cancer Research Centre, University of New South Wales, Sydney, NSW, Australia; 2 School of Women’s and Children’s Health, Faculty of Medicine, University of New South Wales, Sydney, Australia; 3 Electron Microscope Unit, Mark Wainwright Analytical Centre, Chemical Sciences Building, University of New South Wales, Sydney, NSW, Australia; 4 School of Medical Sciences, University of New South Wales, Sydney, NSW, Australia; 5 ARC Centre of Excellence in Convergent Bio-Nano Science and Technology, and Drug Delivery, Disposition and Dynamics, Monash Institute of Pharmaceutical Sciences, Monash University, Parkville, VIC, Australia; 6 Tumor Biology and Targeting Program, Children’s Cancer Institute, Lowy Cancer Research Centre, University of New South Wales, Sydney, NSW, Australia; 7 Australian Centre for Nanomedicine, ARC Centre of Excellence in Convergent Bio-Nano Science and Technology, University of New South Wales, Sydney, NSW, Australia; 8 Department of Chemistry, University of Warwick, Coventry, UK; 9 Australian Institute for Bioengineering and Nanotechnology, The University of Queensland, St Lucia, QLD 4072, Australia

**Keywords:** nanoparticles, targeting, endocytosis, leukemia, flow cytometry, microscopy

## Abstract

**Purpose:**

Directing nanoparticles to cancer cells without using antibodies is of great interest. Subtle changes to the surface chemistry of nanoparticles can significantly affect their biological fate, including their propensity to associate with different cell populations. For instance, nanoparticles functionalized with thiol-reactive groups can potentially enhance association with cells that over-express cell-surface thiol groups. The potential of such an approach for enhancing drug delivery for childhood acute lymphoblastic leukemia (ALL) cells has not been investigated. Herein, we investigate the impact of thiol-reactive star polymers on the cellular association and the mechanisms of uptake of the nanoparticles.

**Methods:**

We prepared fluorescently labeled star polymers functionalized with an mPEG brush corona and pyridyl disulfide to examine how reactivity to exofacial thiols impacts cellular association with ALL cells. We also studied how variations to the mPEG brush composition could potentially be used as a secondary method for controlling the extent of cell association. Specifically, we examined how the inclusion of shorter diethylene glycol brush moieties into the nanoparticle corona could be used to further influence cell association.

**Results:**

Star polymers incorporating both thiol-reactive and diethylene glycol brush moieties exhibited the highest cellular association, followed by those functionalized solely with thiol reactive groups compared to control nanoparticles in T and B pediatric ALL patient-derived xenografts harvested from the spleens and bone marrow of immunodeficient mice. Transfection of cells with an early endosomal marker and imaging with correlative light and electron microscopy confirmed cellular uptake. Endocytosis inhibitors revealed dynamin-dependent clathrin-mediated endocytosis as the main uptake pathway for all the star polymers.

**Conclusion:**

Thiol-reactive star polymers having an mPEG brush corona that includes a proportion of diethylene glycol brush moieties represent a potential strategy for improved leukemia cell delivery.

## Introduction

Acute lymphoblastic leukemia (ALL) is the most common childhood malignancy.^1^ Although current treatment regimens have greatly improved the survival rates of patients, limitations of conventional chemotherapeutics including lack of target specificity, can result in life-long debilitating side effects in more than 60% of survivors.^2^ The use of nanocarriers holds great promise for cancer treatment, yet targeted delivery to cancer cells remains as one of the main challenges of nanomedicine. While immunoglobulins have traditionally been used for the generation of targeted nanoparticles, their clinical translation is often hampered by the suboptimal characteristics of the resulting complexes, including high cost and potential immunogenicity.^3^ Identification of alternative targeting moieties with affinities comparable to antibodies has also proven to be challenging.^4^

Several studies have demonstrated that exploiting the differential expression of cell-surface (exofacial) thiol groups in different cell types can enhance the cellular uptake of peptides as well as nanoparticles.^5^–^8^ The roles of cell surface thiols on the activation and proliferation of lymphocytes have been well documented.^9^,^10^ Moreover, cancer cells have been shown to express higher levels of exofacial thiols, including glutathione and cysteine, compared to their non-transformed counterparts.^11^ However, the targeting efficacy of thiol-functionalized nanocarriers against malignant hematological cells has not been demonstrated previously.

Studies have found that increased exofacial thiols in leukemia may play a vital role in thiol-mediated protection and drug resistance.^12^,^13^ Also, leukemia cells may home to sanctuary sites including the bone marrow (BM) niches, where the highly hypoxic microenvironment provides protection from systemic chemotherapy, thereby increasing the likelihood of relapse.^14^ Reportedly, cancer exofacial thiols may be further upregulated in the hypoxic tumor microenvironment.^15^ However, the impact of the BM hypoxic microenvironment on the expression of leukemic exofacial thiols is not fully understood.

Recently, star polymers have gained considerable attention for use in nanomedicine due to their ease of synthesis and potential to incorporate efficient coupling chemistries.^16^,^17^ The synthesis of star polymers via an arm-first methodology using reversible addition–fragmentation chain transfer (RAFT) polymerization allows the preparation of materials with both reactive sites for functionalization and an antifouling mPEG brush corona.^18^–^20^ Variants of these materials have been explored for the delivery of chemotherapeutic agents including doxorubicin,^20^ siRNA^21^,^22^ as well as MRI contrast agents for imaging^23^ and theranostics.^24^ Star polymers have also displayed prolonged circulation times due to their highly PEGylated nature.^25^

Functionalization of star polymers with thiol-reactive groups can increase their cellular association though potential exchange with cell-surface thiols.^26^ Moreover, thiol functionalization of nanoparticles offers a flexible strategy for the cleavable conjugation of thiol-containing drugs under mild reaction conditions.^27^ To date, studies in this area have employed particles with an mPEG brush corona wherein each mPEG chain (i.e., each “bristle” of the brush) includes 8–9 ethoxy repeat units. Herein, we investigated how incorporating very short ethoxy chain lengths into the star corona might affect both star formation and interactions with cells. To this end, we have prepared star polymers in which a portion of the star arms (carrying thiol-reactive groups) are comprised of diethylene glycol ethyl ether acrylate (DEGA) repeat units. Both homopolymers and copolymers of DEGA have been shown to exhibit different solution properties in water compared to polymers having longer mPEG side chains. For example, solutions of poly(diethylene glycol ethyl ether acrylate) (PDEGA) in water become turbid above ca. 15°C, whereas solutions of poly(oligoethylene glycol methyl ether acrylate) (POEGA) remain soluble up to ca. 92°C.^25^,^28^ As such, star polymers in which a proportion of POEGA arms have been replaced with PDEGA will possess a greater degree of hydrophobicity at physiological temperature compared to star polymers formed purely from POEGA, thereby potentially influencing particle-cell interactions.

Broadly, ALL is categorized as B-ALL or T-ALL based on the origin of lineage.^29^ Compared to immortalized cell lines, ALL patient-derived xenografts (PDXs) in immune-deficient mice represent an orthotopic model of the disease, which retains similar morphology, immunophenotype, and genetic characteristics of the original disease.^30^,^31^ Herein, the cellular association of the star polymers against a panel of B-ALL and T-ALL PDXs was tested. Drug delivery and biological responses of nanocarriers are strongly determined by the mechanism through which they are internalized.^32^,^33^ Therefore, the uptake pathway of the star polymers was analyzed using endocytosis inhibitors and analysis with confocal microscopy and flow cytometry. Finally, we modified Correlative Light Electron Microscopy (CLEM)^34^ for imaging of suspension cells to confirm the uptake and intracellular localization of star polymers.

Our study examines whether thiol functionalization and modification of the oligo (ethylene glycol) (OEGA) corona can improve cellular association and uptake in childhood ALL cells and contributes to the understanding of the internalization pathway of the star polymers to be used for drug delivery.

## Materials and Methods

### Nanoparticle Synthesis

#### Materials

Oligo(ethylene glycol) methyl ether acrylate (OEGA) (Merck, M_n_ = 490 g/mol, OEGA_490_) and diethylene glycol ethyl ether acrylate (DEGA) were purified by percolating over a column of basic alumina before use. Azobis(isobutyronitrile) (Merck, AIBN) was recrystallized from methanol before use. *N,N′*-methylenebis(acrylamide) (Merck, 98%), 2-vinyl-4,4-dimethyl-5-oxazolone (VDM, Polysciences) and Cyanine5 amine (Lumiprobe, Cy5) at the highest purity were used as received. Toluene, chloroform and diethyl ether (Merck Millipore) were used as received. Chain transfer agents (3-(benzylsulfanylthiocarbonylsulfanyl) propionic acid (BSPA) and 2-(pyridine-2-yldisulfanyl) ethyl 2-(((dodecylthio)carbonothioyl)thio)propanoate (PDSD) were synthesized as previously described.^35^

### Synthesis of POEGA-BSPA, POEGA-PDSD and PDEGA-PDSD Linear Arms

Synthesis of POEGA with a terminal benzyl group (POEGA-BSPA) was completed as follows: OEGA_490_ (3.0 g, 6.1 mmol), BSPA (51 mg, 0.19 mmol) and AIBN (3.0 mg, 0.02 mmol) were dissolved in toluene (9 mL) and deoxygenated by sparging with N_2_ (30 mins at 0°C). Polymerization was conducted at 70°C for 4 hrs, after which the reaction was stopped by cooling in an ice bath and exposing to air. Monomer conversion and molecular weight distribution were determined by ^1^H nuclear magnetic resonance spectroscopy (^1^H NMR) and size exclusion chromatography (SEC), respectively. The polymer (POEGA-BSPA) was purified by repeated precipitations in cold diethyl ether and then dried under vacuum. For POEGA-PDSD, the same procedure was employed using OEGA_490_ (3.0 g, 6.1 mmol), PDSD (96.5 mg, 0.19 mmol) and AIBN (3.1 mg, 0.02 mmol). For PDEGA-PDSD the formulation employed was DEGA (3.0 g, 16 mmol), PDSD (251 mg, 0.48 mmol) and AIBN (7.9 mg, 0.05 mmol).

### Synthesis of Core-Crosslinked Star Polymers

A typical synthesis of a core crosslinked star polymer (using the example of a POEGA star with a PDS corona with a VDM functional core (Star-OEGA-PDS) was conducted as follows: OEGA-PDSD linear arm (510 mg, 0.05 mmol, M_n_ = 10,200 g/mol, Đ = 1.2), VDM (13.9 mg, 0.10 mmol), *N,N’-*methylenebisacrylamide (53.9 mg, 0.35 mmol), AIBN (2.5 mg, 0.015 mmol), and toluene (5 mL) were charged to a vial with a magnetic stirrer bar and sparged with N_2_ (30 mins at 0 °C). The polymerization was then conducted at 70 °C for 24 hrs, after which the reaction was stopped by cooling in an ice bath and exposing to air. The polymer was purified by precipitating 5 times from chloroform into chloroform in diethyl ether/(12% v/v) and then dried under vacuum. The star polymers were then characterized using ^1^H NMR and SEC. For mixed composition stars (those incorporating PDEGA arms (Star-OEGA/DEGA-PDS)), the total number of moles of arms was kept constant (0.05 mmol), and the relative amounts of each arm was varied based on desired composition and individual molecular weights. All other details were kept constant.

### Nanoparticles Characterization

Experimental details for the characterization of the star polymers with SEC, ^1^H NMR, and Dynamic Light scattering (DLS), are described in Supplementary materials.

### PDX Culture

Continuous xenografts from children with B-ALL or T-ALL had been previously established in immune-deficient mice as described previously.^30^ PDXs were then harvested from spleen- and BM of engrafted mice with >95% human CD45^+^ by Ficoll gradient centrifugation and cryopreserved at >95% purity. For ex vivo culture experiments, PDX cells were retrieved from cryostorage and thawed at 37°C and washed twice in RPMI1640 media (Merck KGaA, Germany) supplemented with 10% fetal bovine serum (FBS) (Life Technologies) with centrifugation at 500 × g, 5 mins. The cell density and viability were calculated using 0.4% Trypan blue (Sigma-Aldrich) assay. The PDXs were then resuspended at the appropriate cell density in QBSF-60 media (Quality Biological) supplemented with 20 ng/mL Fms-like tyrosine kinase 3 ligand (Flt-3L; ProSpec). Biological replicates were defined as the same passage of the PDX but harvested from different mice. PDX cells used in this study were obtained under approval from the University of New South Wales Animal Care and Ethics Committee (ACEC 16/168B) according to the Australian code for the care and use of animals for scientific purposes (8th Edition 2013).

### Cell Line Culture

Validated stocks of human Jurkat (T-ALL) and Nalm-6 (B-ALL) cell lines were cultured in RPMI1640 media supplemented with 10% FBS. The cell lines were free of mycoplasma contamination. HUVEC (Umbilical Vein Endothelial Cells, CC-2517, Lonza) were cultured in EGM^®^ Endothelial Cell Growth Medium BulletKit^®^ (Lonza) supplemented with 1% Penicillin-Streptomycin (5000 U/mL). Human clonal hepatocyte PH5CH8 cells were kindly provided by Dr. Kyle Hoehm, School of Biotechnology & Biomolecular Sciences, UNSW, and cultured in DMEM media supplemented with 10% FBS. The cells were maintained in a humidified atmosphere (5% CO_2_) at 37°C and, resuspended in fresh media, and allowed to acclimatize for 24 hrs prior to each experiment.

### Cell Viability Assessment

Cell viability was determined using the cell permeable resazurin which is converted to the fluorescent resorufin by viable cells. Jurkat and Nalm-6 cells were seeded in a U-bottom 96-well plate at 2 × 10^5^ cells/mL and exposed to the star polymers at 1–100 mg/L for 48 hrs. Non-malignant human peripheral blood mononuclear cells (PBMCs from the Australian Red Cross Blood Service) and clonal hepatocytes (PH5CH8) were seeded in a U-bottom 96-well plate at 1.5 x 10^5^ and 5 x 10^4^ cells/mL, respectively. Cells were exposed to the star polymers at 32.5–500 mg/L for 24 hrs. Resazurin was added to the plates and the absorbance (excitation/emission 560/590 nm) was measured after 3 hrs using Victor3 1420 Multilabel Counter. PBMCs isolated from 3 different individuals were used in 3 independent experiments. HUVEC cells were seeded in 96 well clear bottom black plate (1 × 10^5^ cells/mL) and exposed to the nanoparticles (31–1000 mg/L) for 24 hrs. Then, the cells were washed twice with phosphate buffered saline (PBS) followed by incubation with 10% Alamar blue (Invitrogen) for 4 hrs and measurement using microplate reader (CLARIOstar, BMG LABTECH). The results were calculated as a percentage of the negative control. Three biological and three technical replicates were used.

### Cellular Association of Star Polymers

PDX cells were seeded at 5 × 10^5^ cells/mL in QBSF-60 media supplemented with 20 ng/mL Flt-3L and exposed to the star polymers for 30 mins at 37°C at 10 mg/L. The samples were then washed three times with PBS (centrifugation at 500 × g, 5 mins) prior to flow cytometry analysis (FACSCanto, BD Biosciences, excitation/emission 650/660 nm). Ten thousand cells were analyzed per sample and the median fluorescence intensity (MFI), normalized against Star-OEGA-Bz and non-treated samples was calculated using FlowJo V10 software (FlowJo, LLC). For the time course studies, Jurkat and Nalm-6 cell lines at 2 × 10^5^ cells/mL were exposed to the star polymers at 10 mg/L for 5–30 mins at 37°C. Following washing with PBS at each time point, the cells were analyzed by flow cytometry as described. All experiments were performed in triplicate.

### Clem

Cells were electroporated with 2*FYVE-GFP to denote phosphinositde-3-phosphate (PI-3-P) enriched early endosomes 24 hrs prior to confocal microscopy. Transfected cells were treated with the star polymers in suspension, adhered to 35 mm MatTek in-plane gridded dishes (MatTek Corporation) coated with 0.01% poly-D-lysine, and fixed in 4% paraformaldehyde. Bright field images and confocal z-stacks were obtained to resolve the alphanumeric coordinate of specific cells. Transmission electron microscopy (TEM) processing was performed by re-fixation of the cells with 2.5% glutaraldehyde in 0.1 M sodium cacodylate buffer (pH 7.4) for 1 hr at room temperature and washed in 0.1 M sodium cacodylate buffer. Post-fixation with 1% osmium tetroxide (w/v) in 0.1 M sodium cacodylate buffer was performed in BioWAVE microwave (Pelco). The cells were serially dehydrated in ethanol (30%, 50%, 70%, 90%, and 100%) and then serially infiltrated with LX112 resin (33%, 66% and 100%) and polymerized to hardness overnight at 60°C. 60 nm ultrathin sections were cut on a Leica UC6 Ultramicrotome (Leica Microsystem) and imaged on a JEOL1400 TEM fitted with an Erlangshen ES500 (Gatan Inc.) camera under the control of Digital Micrograph.

### Endocytosis Pathway Analysis

Jurkat and Nalm-6 cells were seeded at 2 × 10^5^ cells/mL and treated with endocytosis inhibitors. To inhibit caveolin-dependent endocytosis Nystatin (80 µM, 30 mins, Sigma-Aldrich, Australia), and Methyl-β-Cyclodextrin (MβCD) (4 mM, 40 mins, Sigma-Aldrich, Australia) were used. To inhibit clathrin-mediated endocytosis (CME): Pitstop^®^2 (20 µM, 10 mins, Abcam, UK) and. Dyngo^®^4a (30 µM, 30 mins Abcam, UK) were used. To inhibit macropinocytosis: 1 hr treatment with Wortmannin (10 µM Sigma-Aldrich, Australia) or Amiloride (25 µM, Sigma-Aldrich, Australia) was used. The cells were then exposed to the star polymers for 20 mins and thereafter washed three times with PBS before analysis by flow cytometry as described. For confocal microscopy analysis, the cells were seeded at 2 × 10^5^ cells/mL on a FluoroDish™ (World Precision Instruments, Inc.) and imaged with Leica TCS SP8 DLS (Leica Microsystems) using the 63× oil immersion objective. Hoechst 33342 (ThermoFisher Scientific) was used for nuclei staining. All experiments were performed in triplicate.

### Statistical Analysis

The statistical significance of the results was determined using two-way ANOVA with Dunnett’s multiple comparisons. Non-parametric *T* test (Mann–Whitney U) was applied to analyze the difference between the uptake of star polymers in B-ALL and T-ALL cells. The statistical analysis was performed using GraphPad Prism software (GraphPad, CA, USA). The results are presented as the mean ± standard error. A P value ≤0.05 was considered statistically significant.

## Results

### Synthesis and Characterization of Star Polymers

**Figure 1:**
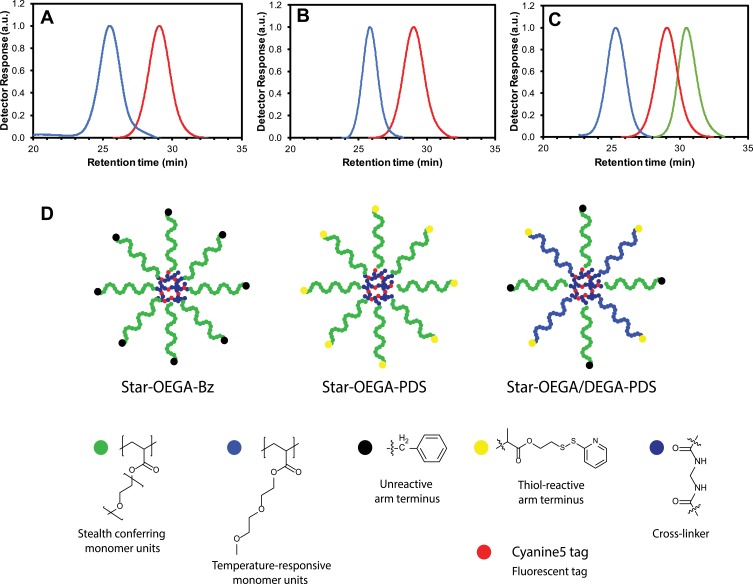
Synthesis of star polymers. (**A–C**) Size exclusion chromatographs of star polymers. (**A**) POEGA stars with unreactive peripheral moieties (“BSPA”) (blue) and POEGA-BSPA arms (red). (**B**) POEGA stars with thiol reactive moieties (“PDS”) (blue) and POEGA-PDSD arms (red). (**C**) POEGA/PDEGA (50/50) stars with thiol reactive groups on the PDEGA arms (“DEG”), POEGA-BSPA arms (red) and PDEGA-PDSD arms (green). (**D**) Schematic of the star polymers. **Abbreviations:** Star-OEGA-Bz, Star polymers incorporating a POEGA corona with BSPA; POEGA, Poly oligo (ethylene glycol) methyl ether acrylate.

Star polymers with varying coronal composition and thiol-reactive peripheral moieties were synthesized via an arm first methodology using RAFT polymerization. Two stars were synthesized incorporating a POEGA corona with either (i) thiol-reactive groups or (ii) non-reactive benzyl groups at the periphery (denoted as Star-OEGA-PDS and Star-OEGA-Bz, respectively (Figure 1). Benzyl-terminated linear POEGA (POEGA-BSPA) was prepared by polymerizing OEGA_490_ in toluene with BSPA, resulting in macromolecular chain transfer agents with benzyl groups at the chain end distal from the thiocarbonylthio moiety (*M_n_* = 11,400 g/mol, *Ð* = 1.22, Figure S1). Synthesis of Pyridyl disulfide-terminated POEGA (POEGA-PDS) was achieved by polymerizing OEGA_490_ in toluene with the chain transfer agent PDSD, yielding polymers with a thiol-reactive group at the periphery (*M_n_* = 10,200 g/mol, *Ð* = 1.19, Figure S2).

These materials were then independently used to prepare core crosslinked star polymers (denoted as “Star-OEGA-Bz” and “Star-OEGA-PDS”) by chain extending with a difunctional crosslinking agent (*N,N’*-methylenebisacrylamide) and a reactive monomer (VDM). VDM was used to provide reactive oxazolone groups within the crosslinked core to introduce a fluorescent label. After purification, core-crosslinked stars with a POEGA corona and either thiol-reactive or inert peripheral groups were achieved. Characterization via SEC confirmed that the residual arm polymer was quantitatively removed and that the molecular weight distribution was unimodal with little evidence of low molecular weight tailing or star-star coupling (Figure 1A for Star-OEGA-Bz *M_n_* = 62,100 g mol^−1^ and *Ð* = 1.25; Figure 1B for Star-OEGA-PDS *M_n_* = 69,100 g mol^−1^ and *Ð* = 1.11). Importantly, the benzyl groups were preserved during the synthesis of the Star-OEGA-Bz star, with the peaks at 7.2–7.3 ppm clearly evident in the ^1^H NMR spectrum of the final purified material (Figure S3). Likewise, the pyridyl disulfide groups were also unaffected by the polymerization process, with the characteristic pattern of peaks at 7.25, 7.85 and 8.5 ppm clearly evident in the spectrum of the purified Star-OEGA-PDS (Figure S4). Analysis by DLS revealed the number average hydrodynamic diameter to be 9 and 8 nm for Star-OEGA-Bz and Star-OEGA-PDS particles, respectively. Successful Cy5 labelling was confirmed by SEC with dual RI/UV/VIS detection, with the SEC trace detected at 646 nm overlapping with that detected by RI (Figure S5).

To examine how changes in the OEGA coating impact on cell association, a third star was prepared incorporating both OEGA and DEGA repeat units in the star corona (denoted as “DEG”). Homopolymers of DEGA are substantially more hydrophobic than homopolymers of OEGA, and typically form turbid solutions in water above 15°C (i.e., they exhibit a so-called lower critical solubility temperature (LCST) of ca. 15°C).^28^ Therefore, star polymers in which a proportion of the POEGA arms are substituted with PDEGA arms would be expected to exhibit some degree of hydrophobic character at 37°C. Moreover, the shorter ethoxy chains might also lead to reduced steric hindrance around the thiol reactive groups at the star periphery. To prepare these POEGA-PDEGA star polymers, homopolymeric PDEGA with a terminal pyridyl disulfide group (PDEGA-PDSD) was first synthesized by polymerizing DEGA in toluene using PDSD as the RAFT agent (Mn = 5000 g mol^−1^, Ð = 1.17, Figure S6). This was then combined with an equimolar amount of POEGA-BSPA in the star formation step with *N,N’*-methylenebisacrylamide and VDM. The polymer was purified and the final composition of DEGA units determined via ^1^H NMR (43 mol%, Figure S7). In contrast to the Star-OEGA-Bz and PDS POEGA-stars, which have either pyridyl disulfide or benzyl groups at the end of the arms, the Star-OEGA/DEGA-PDS particles include both benzyl and pyridyl disulfide groups in the final structure. Importantly, both benzyl and pyridyl disulfide groups were preserved in the formation of the Star-OEGA/DEGA-PDS particles, with the corresponding peaks at 7.2–7.3, 7.85 and 8.5 ppm clearly observable (Figure S7). SEC analysis showed a unimodal distribution with no high or low molecular weight artefacts (Figure 1C, *M_n_* = 92,200 g/mol, *Ð* = 1.14) and DLS revealed a particle size of 8 nm, comparable to the POEGA stars without PDEGA arms. The PDEGA-POEGA 50/50 stars were then successfully fluorescently labeled using Cy5.

### Impact of Star Polymers on Cell Viability

**Figure 2:**
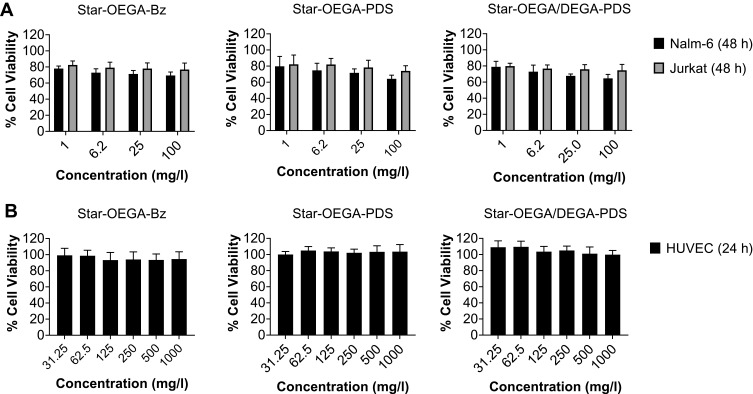
Impact of star polymers on cell viability. (**A**) Jurkat and Nalm-6 cells (**B**) HUVEC cells were exposed to the star polymers for 24 hrs and 48 hrs, respectively. The cell viability was measured using the resazurin assay.

As drug carriers, nanoparticles must be biocompatible. Jurkat and Nalm-6 cells were exposed to the star polymers for 48 hrs (Figure 2A). At the lowest (1 mg/l) and highest (100 mg/l) concentration, there was 20 and 35% reduction in cell viability, respectively. To assess the impact on non-cancer cells, HUVEC, human PBMCs and clonal hepatocytes (PH5CH8) were exposed to the star polymers for 24 hrs at higher concentrations (31.25–1000 mg/l) and negligible reduction on cell viability was observed, indicating good tolerability of the cell lines to the star polymers (Figures 2B and S8).

### Functionalized Star Polymers Exhibit Enhanced Association with PDXs

**Figure 3:**
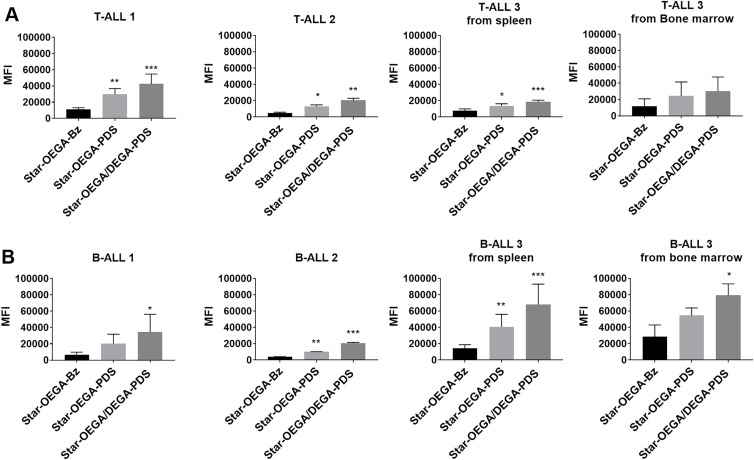
Thiol-reactive star polymers display enhanced association with patient derived xenografts (PDXs). (**A**) PDXs were generated from mononuclear cells from pediatric ALL biopsies (B- and T-ALL) inoculated in immunodeficient mice. Three PDX samples from each subtype harvested from spleens, and one matched sample from bone marrow, were exposed to the star polymers for 30 mins. (**B**) Cellular association was determined by flow cytometry and represented by a median fluorescence intensity (MFI) normalized against control Star-OEGA-Bz. * p < 0.05, ** p < 0.01. *** p < 0.001.

Cancer cells overexpress exofacial free thiols.^12^,^13^ Therefore, we first confirmed the effect of thiol functionalization on the cellular association of star polymers with a panel of B-ALL and T-ALL PDXs harvested from the spleens of immune deficient mice (Figure 3). Both thiol-reactive star polymers (Star-OEGA/DEGA-PDS and Star-OEGA-PDS) displayed a statistically significant enhanced cellular association to both subtypes compared to Star-OEGA-Bz, as indicated by MFI per cell (Figure 3). Star-OEGA/DEGA-PDS had the highest uptake in all PDXs. The thiol-reactive star polymers also exhibited a significantly higher association with the B-ALL compared to T-ALL PDXs (Figure S9A and B). There was however no difference in the association of Star-OEGA-Bz with either subtypes (Figure S9C). The presence of leukemic blasts can lead to an increase in the hypoxia of the BM niche.^36^ Since exofacial thiols may be upregulated in the hypoxic tumor microenvironment,^15^ we tested the cellular association of the star polymers in matched B-ALL and T-ALL PDX samples collected from the BM and spleens. Overall, the BM lymphoblasts exhibited similar association to the star polymers as those observed in spleen lymphoblast (Figure 3).

### Functionalized Star Polymers Exhibit Enhanced Association with ALL Cell Lines

**Figure 4:**
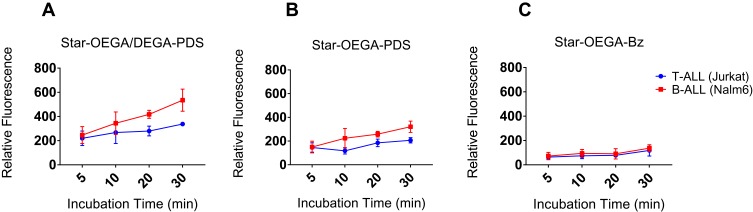
Thiol-reactive star polymers display enhanced association with Jurkat and Nalm-6 cell lines. The cells were incubated with the star polymers at 10 mg/l for up to 30 mins at 37°C. (**A**) Star-OEGA/DEGA-PDS exhibited the highest association and as early as 5 mins followed by (**B**) Star-OEGA-PDS and (**C**) Star-OEGA-Bz. The B-ALL cell line (Nalm-6) exhibited higher association with the thiol-reactive star polymers compared to Jurkat (T-ALL) cell line. For Star-OEGA-Bz with unreactive groups at the periphery the association was similar in both cell lines. All experiments were performed in triplicate.

The cellular association of the star polymers with cancer cell lines Jurkat (T-ALL) and Nalm-6 (B-ALL) was performed to compare the findings with PDXs. Contrary to PDXs, cell lines are cultured in media containing serum which may impact cellular interactions with the star polymers. The cellular association of all star polymers increased with time. The Star-OEGA/DEGA-PDS had the highest association followed by Star-OEGA-PDS (Figure 4A and B). However, there was little cell association with Star-OEGA-Bz in both cell lines (Figure 4C). Overall, the cell lines gave similar results to the PDXs. The Nalm-6 (B-ALL) cell lines exhibited higher association with both thiol-reactive star polymers compared to Jurkat (T-ALL). For Star-OEGA-Bz on the other hand, the cellular association was similar between Nalm-6 and Jurkat.

### Cellular Uptake of the Star Polymers

**Figure 5:**
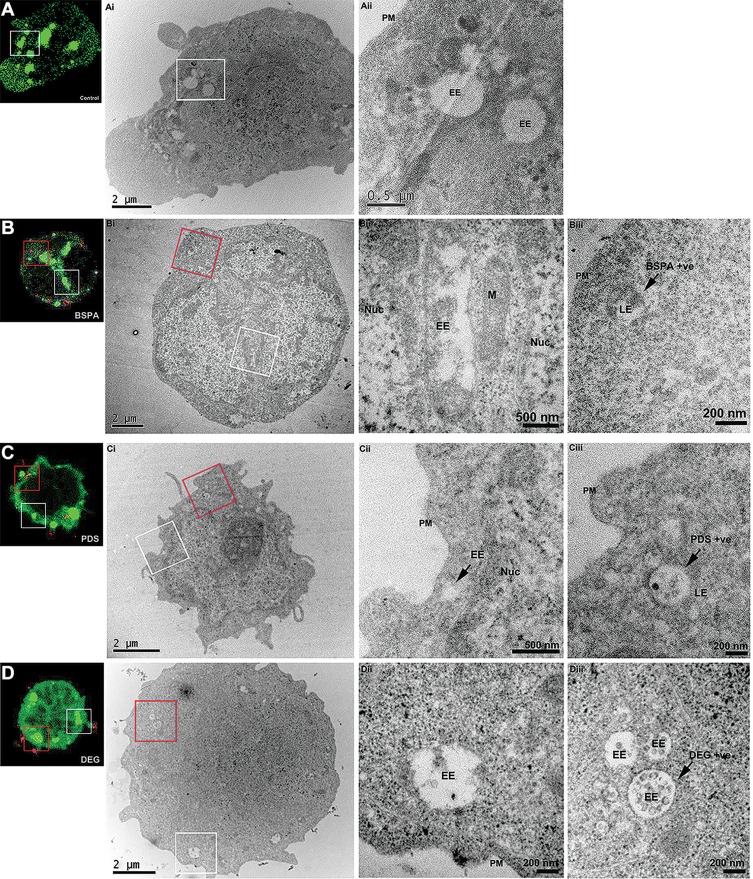
Correlative light and electron microscopy (CLEM) reveals the endosomal accumulation of star polymers. Nalm-6 cells were transfected with the early endosomal marker 2*FYVE-GFP and treated with the star polymers for 20 mins. Left panels: Nalm-6 cells imaged by confocal microscopy and (i–iii) corresponding electron micrographs of the same cell. White box denotes correlated early endosomes from the confocal Z-stack and visualized in further detail at (i) low-magnification and (ii) high-magnification. Red box highlights the correlated nanoparticles-positive endosomes (iii). (**A**) Control untreated Nalm-6 cell denote early endosomes highlighted in green without treatment of star polymers. (**B**) cells treated with Star-OEGA-Bz demonstrated abundant early endosomes and rapid accumulation of Star-OEGA-Bz within a distinct population of endomembranes identified as late endosome/lysosome by high-resolution morphometric analysis. (**C**) Star-OEGA-PDS rapidly accumulated within late endosomes/lysosomes. (**D**) Star-OEGA/DEGA-PDS stars trafficked in early endosomes after 20 mins uptake. **Abbreviations:** Nuc, nucleus; EE, early endosomes; PM, plasma membrane; M, mitochondria; LE, late endosome/lysosome.

CLEM allows for the coupling of fluorescence microscopy with TEM to resolve uptake of nanoparticles into individual organelles within specific cells. This facilitates the localization of nanoparticles in high-resolution even in the absence of a specific fluorescent marker for individual cellular compartments. To characterize the internalization and trafficking of star polymers, Nalm-6 cells were transfected with 2*FYVE-GFP to label PI-3-P enriched domains within early endosomes.^34^,^37^ We demonstrated that the fluorescence of the 2*FYVE-GFP marker correlated within endosomes in untreated control cells (Figure 5A). Star-OEGA-Bz quickly trafficked out of the PI-3-P positive early endosomes and localized to membrane-encapsulated structures that morphologically resembled late endosomes/lysosomes (Figure 5B). Similarly, Star-OEGA-PDS were rapidly sorted out of early endosomes and accumulated within morphologically identifiable late endosomes/lysosomes (Figure 5C). Finally, Star-OEGA/DEGA-PDS were also efficiently internalized but trafficked through the endosomal system more slowly than Star-OEGA-PDS and Star-OEGA-Bz particles. After 20 mins of incubation Star-OEGA/DEGA-PDS was still observed within 2*FYVE-GFP decorated early endosomes (Figure 5D). These observations confirm that the star polymers traffic through the canonical endosomal system and that coronal composition impacts on intracellular trafficking.

### Endocytosis Mechanism of Star Polymers

**Figure 6:**
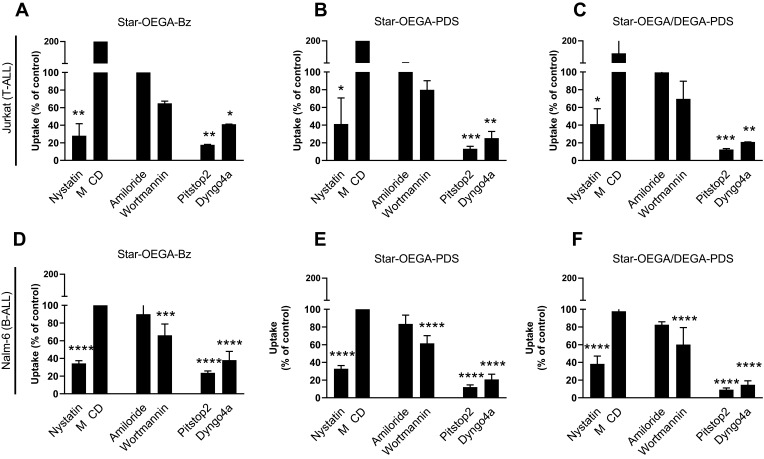
Endocytosis pathway of star polymers. Jurkat and Nalm-6 and cells were treated with endocytosis inhibitors before exposure to the star polymers (10 mg/l for 30 mins) and analysis by flow cytometry. (**A**–**C**) In Jurkat cell lines endocytosis was blocked by cellular treatment with, Nystatin, Pitstop2, and Dyngo4a. (**D**–**F**) In Nalm-6 cell lines endocytosis was blocked by cellular treatment with Nystatin, Pitstop2, Dyngo4a, and Wortmannin. Data represent the percentage of nanoparticles uptake in cells treated with endocytosis inhibitors to that of the control group without treatment. * p < 0.05, ** p < 0.01. *** p < 0.001 **** p < 0.0001. Methyl-β-cyclodextrin (MβCD).

Endocytosis is divided into phagocytosis (specific to macrophages and neutrophils) and pinocytosis including CME, caveolin-mediated endocytosis, macropinocytosis, and several clathrin independent endocytosis pathways.^38^ As not all inhibitors may work on all cell types, we employed two inhibitors per pathway with different mechanisms of action to ensure efficient inhibition.^39^ Also, as inhibition of one pathway may activate other internalization routes, a short exposure time to star polymers was used.^40^ The flow cytometry analysis showed that none of the inhibitors resulted in complete inhibition of endocytosis, indicating a contribution from several pathways (Figure 6A–F). The uptake of the star polymers in both cell lines was significantly reduced with Pitstop2 which specifically binds to the β-propeller N-terminal domain of clathrin heavy chain thus inhibiting the assembly of clathrin coat and CME.^41^ Also, treatment with Dyngo4a inhibited the uptake of the star polymers in both cell lines suggesting uptake through dynamin dependent endocytosis pathways. Among the inhibitors of caveolin-dependent endocytosis, only Nystatin was effective in inhibiting uptake of the star polymers. The expression of caveolin in lymphocytes is still unclear,^42^ yet as they are abundant in lipid rafts, cholesterol depleting inhibitors including Nystatin can indirectly inhibit caveolae uptake but can also impact CME. Wortmannin also reduced uptake in both cell lines but was only statistically significant in Nalm-6. Although wortmannin was chosen as an inhibitor of macropinocytosis, it acts by aggregation and stabilization of clathrin-coated pits at the plasma membrane^43^,^44^ and thus, may impact CME as well. Amiloride, a specific inhibitor of micropinocytosis^45^ did not inhibit uptake. Confocal microscopy images of the cells pretreated with the inhibitors followed by exposure to the star polymers confirmed the results of flow cytometry analysis (Figure S10). Together these data suggest uptake through dynamin-dependent CME.

## Discussion

We examined whether the naturally present exofacial thiols that are upregulated in cancer cells,^11^,^46^ can be exploited to enhance the cellular association and uptake pathway of thiol-reactive star polymers. The preparation of nanoparticles with an mPEG brush corona has been investigated by our group and others, showing the platform to be suitable for incorporating a wide array of functional moieties. Herein, we have extended our previous investigations to include variations in the composition of the corona. Specifically, by including a second arm material (PDEGA-PDSD) into the star synthesis, we have been able to prepare materials that include not only mPEG brushes with 8–9 ethoxy repeat units, but also much shorter pendants with only two ethoxy groups in the chain. Moreover, the reported methodology allows simple variation to the corona composition, potentially enabling the cell uptake to be tuned for specific applications. Although homopolymeric PDEGA is insoluble at physiological temperature (due to its LCST of ca. 15°C^25^), incorporating PDEGA arms into a star structure which also includes POEGA arms ensured that the materials remained well dispersed even at 37°C. Moreover, using two types of arm, each having a different hydrophilicity, provides a novel approach for tuning the hydrophilic/hydrophobic balance of the star polymer corona.

Endothelial cells covering the lumen of blood vessels are responsible for regulating a myriad of vital physiological functions and serve as the first site of contact for nanoparticles entering the blood. Therefore, the assessment of the toxicity of nanoparticles on endothelial cells provides important information about the potential side effects of nanoparticles.^47^ There was no reduction in viability of HUVEC cells exposed to the star polymers even at high concentrations. The star polymers were also well tolerated by ALL cells even after 48 hrs agreeing with previous findings on the biocompatibility of thiol-reactive nanoparticles.^48^

As these nanoparticles were proposed to treat hematological malignancies, we demonstrated the biocompatibility of these star polymers with normal human PBMCs. Furthermore, we also showed that the star polymers exerted limited cytotoxic effects on the non-malignant hepatocyte cell line (PH5CH8), which is critical as one of the major organs where nanoparticles can accumulate during transfer through the bloodstream is the liver.

The thiol-reactive star polymers had a significantly high association with both B and T ALL PDXs compared to Star-OEGA-Bz, an observation that was confirmed in the cell lines. The B-ALL PDXs as well as cell line (Nalm-6) had higher association with the thiol reactive star polymers compared to T-ALL PDXs and cell lines. However, there was no significant difference between the cellular association of Star-OEGA-Bz with B and T-ALL cell lines and PDXs. Reportedly, B cells naturally express higher levels of exofacial thiols,^9^ which could explain the higher association with thiol-reactive star polymers compared but not Star-OEGA-Bz. Our findings also highlight the role of the overexpressed free thiols in malignant hematopoietic cells and other pathological states. Indeed studies have shown that upon infection, T-cells upregulate exofacial free thiols to regulate cell function and prevent exofacial proteins overoxidation.^49^

We also examined whether lymphoblasts harvested from the BM would exhibit higher association with the thiol-reactive star polymers. However, upon normalization against Star-OEGA-Bz, similar findings to those of PDX cells from spleens were observed, potentially due to the loss of exofacial thiols during the harvesting and cryopreservation procedures. Conversely, the extent of increase of exofacial thiols on cells from the BM may not be enough to impact association with the nanoparticles.

To characterize the trafficking of nanoparticles through the endosomal pathway by fluorescence imaging alone, it is necessary to express highly specific fluorescently-tagged markers of each maturing compartment and co-localize these markers with the differentially tagged nanoparticles. Often, this requires over-expression of fluorescently-tagged proteins, such as Rab proteins, which can severely impact the dynamics of endosomal function.^37^ Therefore, we expressed a non-functional lipid probe, 2*FYVE-GFP, that passively associates with PI-3-P lipid domains enriched within early endosomes.^34^ Our observations demonstrated that Star-OEGA-Bz, Star-OEGA-PDS and Star-OEGA/DEGA-PDS quickly trafficked through the early endosome into downstream compartments. To better characterize this trafficking, we employed a fluorescently-tagged lipid probe of phosphatidylinositol-3,5-biphosphate (PI-3,5-P_2_), 2*ML1N-GFP, to denote late/endosomes lysosomes.^50^ In Nalm-6, PI-3,5-P_2_ was lowly abundant and not clearly defined at late endosomes/lysosomes (data not shown). Therefore, we lacked a specific marker for late endosomes. TEM, unlike confocal microscopy, has the resolving power to localize nanoparticles within a morphologically definable cellular organelle, but the application here was limited as star polymers have a similar atomic mass to that of the surrounding cell (primarily carbon) and, consequently, can be difficult to distinguish. Therefore, CLEM was performed as it exploits fluorescently tagged cellular compartments as fiducial markers such that the position of the nanoparticles in the cell, by fluorescence, can be correlated with morphology of these same compartments by TEM. We confirmed the efficient uptake and endosomal internalization of the star polymers. Interestingly, despite having the highest cellular association in both PDXs and cell lines and as early as 5 mins, Star-OEGA/DEGA-PDS trafficked through the endosomal system slower than the other nanoparticles, potentially due to enhanced affinity to the cell membrane,^51^ as well as the presence of thiol functional groups.

The fundamental step in designing efficient nanocarriers is understanding their specific uptake pathway as it will have a direct impact on the intracellular localization and efficiency of drug delivery. The physicochemical properties of nanoparticles, including size, surface functionalization, as well as cell type may govern cellular interactions.^52^ Also, reportedly, endocytosis is dysregulated in cancer and trafficking can be disturbed downstream of oncogenes.^53^,^54^ Here, the predominant uptake pathway for the star polymers, irrespective of functionalization or cell type, was dynamin-dependent CME. The CME pathway is involved in an array of vital cellular processes and is initiated by the formation of clathrin-coated pits resulting in the loading of cargo into the cell membrane and subsequent internalization. The cargo loaded endosomes then traffic along the cytoskeleton to fuse with early or sorting endosomes, prior to either being recycled back to their original membrane domain or being transported along the endo-lysosomal pathway for degradation in the lysosomes.^55^ Therefore, this pathway is optimal for nanomedicines intended for drug accumulation and release in lysosomes.^56^,^57^ Our data showed that the CME was dynamin dependent, a GTPase involved in CME and caveolin-mediated endocytosis that forms a coil-like structure around the neck of newly formed endosomes enabling release of the vesicle.^58^ Although the size of the star polymers was also suitable for caveolin-mediated endocytosis,^59^,^60^ the expression of caveolin in lymphocytes is still unclear^61^ and therefore a definitive conclusion could not be made. A previous study also showed that while single and mixed endocytosis inhibitors did not efficiently inhibit the uptake of maleimide-modified liposomes, a marked decrease in uptake was observed when the cellular thiols were pre-blocked, suggesting a thiol-mediated membrane trafficking, including endocytosis.^62^

## Conclusion

In conclusion, we have shown that thiol-reactive star polymers functionalized with short ethoxy-containing moieties increases cellular association without altering the uptake pathway, but the rate of intracellular trafficking may be affected. Further studies are needed to completely understand the effect of the star polymers in vivo and after conjugation with drugs. However, this study represents the first step in revealing the potential application of thiol-reactive star polymers in the passive targeting of malignant hematopoietic cells.

## Abbreviations

ALL, acute lymphoblastic leukemia; BSPA, 3-(benzylsulfanylthiocarbonylsulfanyl) propionic acid; CME, clathrin-mediated endocytosis; CLEM, Correlative Light Electron Microscopy; DEGA, diethylene glycol ethyl ether acrylate; MβCD, Methyl-β-Cyclodextrin; OEGA, Poligo(ethylene glycol) methyl ether acrylate; PBMCs, peripheral blood mononuclear cells; POEGA, Poly oligo(ethylene glycol) methyl ether acrylate; PDSD, 2-(pyridine-2-yldisulfanyl) ethyl 2-(((dodecylthio)carbonothioyl)thio)propanoate; PDX, patient-derived xenograft; Star-OEGA/DEGA-PDS, star polymers incorporating a POEGA corona with DEGA and PDSD; Star-OEGA-Bz, star polymers incorporating a POEGA corona with BSPA; Star-OEGA-PDS, star polymers incorporating a POEGA corona with PDSD.
